# Dedicated Observers for Sensors Fault Detection and Diagnosis in Real Time for Bioreactors

**DOI:** 10.3390/s26041095

**Published:** 2026-02-08

**Authors:** Patricia Meneses-Martínez, Iraiz González-Viveros, Patricio Ordaz, Ricardo Aguilar-López, Pablo Antonio López-Pérez, Juan Luis Mata-Machuca

**Affiliations:** 1 Research Center on Technology of Information and Systems, Autonomous University of Hidalgo State, Pachuca 42184, Mexico; me477772@uaeh.edu.mx (P.M.-M.); iraiz_gonzalez@uaeh.edu.mx (I.G.-V.); jesus_ordaz@uaeh.edu.mx (P.O.); 2 Departamento de Biotecnología y Bioingeniería, Centro de Investigación y de Estudios Avanzados del Instituto Politécnico Nacional (CINVESTAV-IPN), Unidad Zacatenco, Ciudad de México 07360, Mexico; raguilar@cinvestav.mx; 3 Escuela Superior de Apan, Universidad Autónoma del Estado de Hidalgo, Carretera Apan Calpulalpan km. 8, Apan 43900, Mexico; 4 Departamento de Tecnologías Avanzadas, Unidad Profesional Interdisciplinaria en Ingeniería y Tecnologías Avanzadas (UPIITA), Instituto Politécnico Nacional, Ciudad de México 07340, Mexico

**Keywords:** abrupt failures, bioreactor, fault diagnosis, diagnostic metrics, state estimation

## Abstract

Due to the increasing demand for greater safety and ease of scale bioprocessing, fault detection and diagnosis (FDD) is becoming an effective method to avoid breakdowns and disasters. Therefore, this work focuses on developing a dedicated observer-based fault diagnosis for nonlinear systems. To solve this, the FDD scheme is needed to make it perform satisfactorily even in a faulty situation. A case study on bioethanol production is proposed to illustrate and demonstrate the proposed techniques in real time. Single faults and different sensor faults are considered. The effectiveness of the proposed model is proved by comparing its performance obtained by simulation with the experimental data. In order to supervise the change of the possible faulty parameter, robust adaptive full-order observers that focus not only on the state estimation but also on the parameter change are applied to the considered bioreactor. In order to achieve the desired outcome of sensor fault detection, we propose a residual evaluation function, given by the root-mean-square (RMS) value of the residual and a practical threshold for the bioreactor. Experimental results show that sensor faults can be well diagnosed by the proposed observer-based FDD method. The precision, recall rate, and overall accuracy of three diagnostic metrics for abrupt failures were compared. The diagnostic approach was successful, achieving an overall accuracy rate of over 90% for each of the three abrupt failure scenarios in every sensor. Finally, even if the biomass or $CO_{2}$ sensors fail, the FDD system can reconstruct the substrate and ethanol dynamics that are typically quantified offline in bioprocesses in real time.

## 1. Introduction

In the bioprocess industry, fault detection and diagnosis (FDD) plays an essential role, as it detects faults and their causes in the early stages of development. This allows controllers to make appropriate decisions to tolerate component malfunctions, preventing minor failures from becoming severe ones that affect the quality of the final product, result in irreparable energy and material expenses, or, in the worst case, cause human losses. Monitoring is a concept that encompasses the detection and diagnosis of failures within a process. However, these actions present a challenge in the bioprocess industry because these are nonlinear systems with complex dynamics. It is difficult to discriminate between disturbances and failures under a wide range of operating conditions [1].

The FDD process has three fundamental stages: fault detection, isolation, and estimation. The first stage involves determining whether the system is operating under abnormal conditions. Once a fault is detected, the next stage begins to determine where it occurred: in the system, an actuator, or a sensor. In addition, the defective element is located within all field devices. The final stage of the fault estimation provides information about the magnitude, behavior, and nature of the fault. Accurate failure estimation requires knowledge of its occurrence and location. Therefore, accurate failure estimation plays a relevant role in fault-tolerant control systems (FTCSs), which execute control actions to maintain system performance and stability in the event [2]. One of the great challenges of the bioprocess industry is achieving a favorable environment for the development and growth of microorganisms (biomass) in bioreactors, and consuming enough substrate to produce the desired final product with high performance and productivity. Biotechnological processes are dynamic and involve continuous changes in physicochemical environmental conditions. Real-time monitoring is crucial for securing the most favorable conditions, so strengthening the tools that guarantee this work is essential [2,3].

A state observer is a dynamic system that can be either deterministic or stochastic [4]. It has the ability to reconstruct state variables of a system that are inaccessible because they cannot be directly measured by a physical sensor. State observers are designed based on a mathematical model and available measurements in the plant [5].

For the design applied to this case study, the system model must have the property that it is completely observable [6]. It constitutes a mathematical duplication of the system whose inputs are the inputs of the system and the measurements available in the measuring instruments. It also presents a signal that represents the difference between the measured system and the observer’s output (estimation error) [7]. In Table 1, a brief review about applications of observers in a class of bioprocess is presented.

On the other hand, the cellulosic bioethanol production process by alcoholic fermentation involves four consecutive stages: pretreatment of the lignocellulose material, enzymatic hydrolysis of the pretreated material, hydrolyzate fermentation, and separation processes [12]. Numerous works have been performed that describe the fermentation process in bioreactors for the production of ethanol from lignocellulosic material. This is the case of [12], in which wheat straw is used as a substrate, which is a material rich in glucose, acetic acid, lignin, among other compounds. A yeast strain that consumes glucose and xylose simultaneously is used, and the productivity of the process is derived from the rate at which the latter is consumed. Furfural is an important yeast inhibitor in this case, so its concentration must be kept low throughout fermentation [13]. Another substrate inhibitor is acetic acid, but its effect can be regulated by pH. Therefore, pH becomes the primary control variable. Since fermentation takes place under anaerobic conditions, it is important to monitor oxygen concentration, as its presence decreases the ethanol yield. In addition, it is important to monitor the carbon dioxide concentration and ethanol as the desired product. All of these variables can be monitored in real time by direct measurement or indirect modeling techniques [13].

Vibrational spectroscopy (UV–VIS, NIR, MIR, and Raman spectroscopy) is a widely used method to monitor the alcoholic fermentation process. This set of analytical techniques allows for the rapid detection of components within the fermentation process without the need to take samples. One of the main challenges is the large quantity of solid particles, such as lignin and biomass, that are suspended in the medium and interfere with light, causing reflection and dispersion and limiting vibration transmission. However, vibrational spectroscopy methods, mainly attenuated total reflectance (ATR) and diffuse reflectance, do not depend on light transmitted through the medium, but rather on reflected or backscattered light. This makes these methods useful for the online monitoring of alcoholic fermentation [12].

In [4], infrared spectroscopy (NIRS) methods were used to estimate the specific production rate of the by-products, using online measurements of biomass concentration. To estimate the concentration of by-products, high-pressure liquid chromatography (HPLC) was applied. HPLC is a method used to separate chemical compounds employing a liquid mobile phase and a solid stationary phase. A detector then recognizes the retention time of each chemical compound and determines the concentration by the amplitude of the peak reached. The glucose concentration was measured using HPLC, as well as the metabolic by-products acetate, lactate, formate, and ethanol [14].

In general, contributions in the area of FDD methods are scattered in the technical literature and are so broad that it is already an established field in control engineering. The most basic methods date back to the end of the 19th century, when process instrumentation was implemented [15]. Supervision techniques evolved to greater maturity and complexity with the advent of computers in the mid-20th century. Since the early 1970s, a significant amount of knowledge has been accumulated on model-based fault diagnosis. However, it was not until late 1997 that a book presenting this topic in a unified framework was written, since there was no “common language” among researchers using different terminologies.

In [13], the implementation of soft sensors for bioprocesses with the *Pichia pastoris* strain is described. Furthermore, the importance of monitoring the variable biomass concentration is highlighted as it is the most important in this type of bioprocess, having a direct effect on the expression level of the recombinant protein.

In [16], a control system based on fault detection and diagnosis uses robust transition structures for processes with different operating regimes. These structures are characterized by candidate controllers and a library of models for each operating regime. Structures also have a transition supervisor that decides which candidate controller is best to maintain the process and when to place it in the process feedback loop [17]. In the detection stage, errors are calculated based on the difference between the measured values and those obtained by the fault-free model. For the diagnosis stage, deviations are detected in sensors taking into account the linear relationship between measured value and real value (in voltage) and in actuators according to the linear relationship between the control signal and the valve flow. Deviations from the measured value occur with respect to the representative line of a typical instrument and result in alterations to the modeled device’s components [18].

In the field of biochemical engineering, an intensified bioreactor is a multidisciplinary device that combines requirements of one hybrid unit for safer operating conditions and lower cost, as well as energy waste. On the other hand, ref. [19] performs a scheme to detect and diagnose failures in sensors incorporated into a *batch* reactor based on fuzzy observers using sliding modes. A generalized scheme observer bank is developed to detect additive and intermittent faults in temperature and pH sensors. Finally, residuals are used for decision making by establishing detection thresholds.

Other works, such as [20,21], were consulted and form part of the background to this research, which employ FDD through the use of estimation methods. However, undesirable failures, such as sensor malfunction, complete failure, polarization failure, drift failure, and precision decline failure, still pose a great threat to the intensification and control of bioreactors.

Different methods are recorded in the literature to monitor critical quality attributes and process parameters [22,23]. We will analyze the methods in line, in situ, on line, and off line. To achieve an efficient FDD system in any industrial process, it is crucial to maintain correct control and monitoring of the critical variables involved. These parameters can be grouped into physical, chemical, and biological. This is a simple, practical approach in which each observer receives input and output signals from a single sensor. Therefore, the number of observers must be equal to the number of sensors. In order to implement this approach, the system must be fully observable from all state variables. Each observer generates a vector of estimated states that can be used to generate residuals by comparing them with the actual output of the fully coupled process in the observer bank.

However, most real-time fault detection and diagnosis techniques in bioprocesses currently rely solely on data from one or more sensors. The mentioned methods analyzed neither simultaneous nor sequential failures in multiple sensors, such as abrupt failures. To address these limitations, we propose in this work an FDD scheme that detects abrupt failures for a combination of sensors by analyzing the response of indices generated from the fusion of residues from the biomass and $CO_{2}$ sensors with respect to the estimator, using FDD to reduce noise and improve the accuracy of fault detection through sensor fusion. Moreover, it is important to note that, in the event of a failure of an input sensor (*X*, $CO_{2}$) for the observer, the system will still be able to estimate using the remaining sensors. This is advantageous if the objective is to take a control action. Abrupt failures are modeled as sudden stepwise deviations, whereby the component value changes abruptly from its nominal value to an unknown one. It is also important to note the real-time estimation of substrate and ethanol, as this avoids the need for specialized offline equipment for quantification. The bioreactor can monitor the process even in the event of sensor failure using low-cost sensors, justifying its application in real-life scenarios given that biomass is a precursor to the reaction and $CO_{2}$ is a product of cellular metabolism present in bioprocesses. The proposed robust adaptive observer approach for fault diagnosis (FDD) in the bioreactor has clear and precise advantages over the literature. In contrast with observers based on classical (fixed) models that only detect the fault, the adaptive nature of this design allows for real-time estimation of the change in the faulty parameter, providing a more in-depth diagnosis. Its robustness ensures high accuracy (greater than 90%) by mitigating the impact of noise and uncertainties inherent in the nonlinear process. Furthermore, this approach surpasses purely data-driven (ML) techniques by offering high interpretability based on the physical model and achieving advanced functionality for reconstructing key variables (soft sensing), such as ethanol and substrate, even when biomass or $CO_{2}$ sensors fail, thus guaranteeing operational continuity and bioprocess control. This contribution can be summarized as follows:

- In this paper, laboratory kinetics were developed that allowed us to analyze the behavior of the plant on a semi-pilot scale and propose a mathematical model that represents the kinetics of substrate, biomass, carbon dioxide, and ethanol consumption in the batch fermentation process. It was essential to perform the observability analysis for the proposed model. Based on the criterion of the observability matrix from the measurement of the biomass concentration, taking it as the output of the system, it is possible to guarantee the complete observability of the state vector.
- The combined technique presented here (turbidity sensor and $CO_{2}$) will add great value to the characterization of the process and allow the development of control algorithms, especially for monitoring of bioreactors in real time.
- Subsequently, a real-time fault diagnosis scheme is tested, based on a bank of adaptive observers with the use of the Matlab R2024b^®^ platform. In this stage of the mechanism testing, the signals from the turbidity and $CO_{2}$ sensors are subjected to artificially induced failures using step, ramp, and pulse train signals. It is observed that the FDD scheme manages to detect, isolate, and know the magnitude of the failure in real time by generating estimation residuals for each observer based on DOS.
- These new monitoring technologies, when combined with mathematical models and software tools, are challenging for intelligent process control, performance improvement, and prediction, without the need for invasive sampling or system intervention. In this sense, this work shows an alternative for Industry 4.0 to improve state-of-the-art fault diagnosis technologies and their application in the context of monitoring alcoholic fermentation bioprocesses.

## 2. Methodology

**Figure 1:**
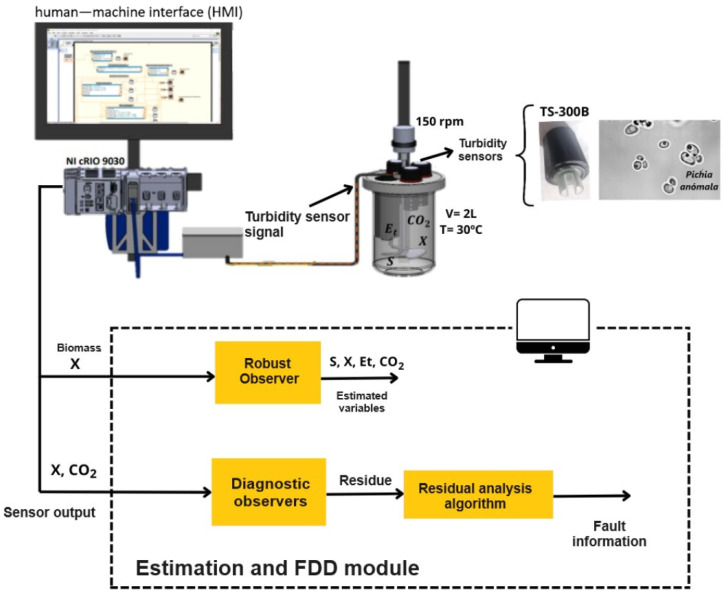
A monitoring system based on a state observer coupled with a fault diagnosis mechanism.

**Figure 2:**
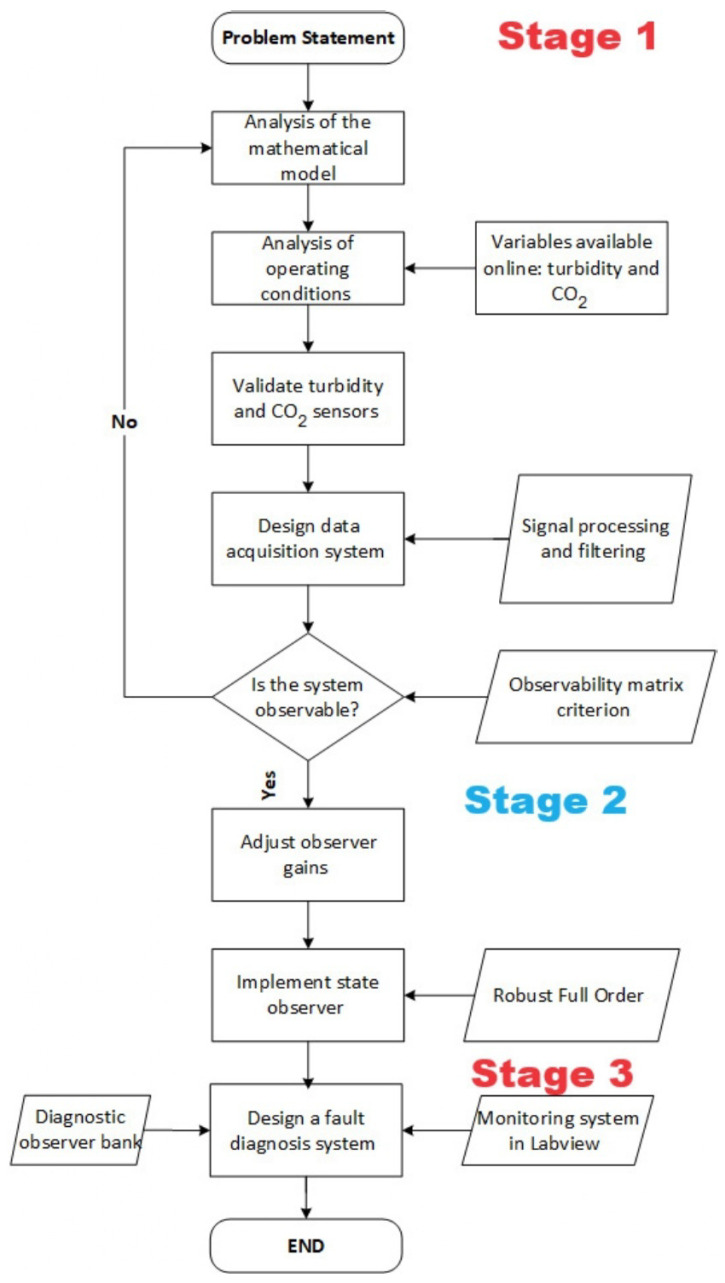
Methodology for real-time detection and diagnosis of sensor faults.

Figure 1 and Figure 2 show the general methodology followed in this case study. The system is based on a 2 L capacity bioreactor made of stainless steel. Sensors are installed in the bioreactor to measure level, temperature, pH, hydrogen, and biomass. The biomass measurement is carried out with a low-cost turbidity sensor, which is very important since it serves as input to the observer who estimates the variables that cannot be measured online, such as substrate and ethanol. In this way, the monitoring system will be based on signals coming from sensors that are previously conditioned (amplification and filtering); additionally, the estimated state variables obtained from the correct measurement provided by the turbidity sensor are considered.

In these types of applications based on state observers, it is assumed that the input signal to the observer is correct, and therefore, the estimation is assertive. However, in real life, this is not the case; measuring instruments can malfunction and have additive failures in their output. In the case of sensors, failures occur due to calibration errors, drifts, short circuits, or false contacts.

To start up the system with the fault diagnosis mechanism, a turbidity sensor (TS300B) was used to measure biomass, while the Vernier $CO_{2}$ sensor was set to measure $CO_{2}$ concentrations ranging from 0 to 100,000 ppm. The partial pressure in the bioreactor airspace was measured at 10 min intervals over a period of 1 week to determine the $CO_{2}$ concentrations and My RIO 6008 OEM data acquisition card and the coir9030 embedded system. First, signal acquisition is performed using the data acquisition card to test the algorithm in Matlab^®^. Finally, the run is performed in real time with the embedded system and the industrial rack, using the environment Labview 2024 Q1™ and the Real Time 2024 Q1 package.

In this section, a more illustrative explanation of the operation of the monitoring system for the detection and diagnosis of sensor failures will be given. For this, 3 we will use the Labview™ Real Time 2024 Q1 interface, which allows for establishing a serial communication protocol with the NI cRIO-9030 controller, where the acquisition and conditioning of sensor signals is carried out. A vision of the entire monitoring system is shown that involves the monitoring of variables that cannot be obtained online, through estimation with real-time measurement of the biomass concentration obtained by two turbidity sensors located as physical redundancy. In addition, the detection and diagnosis mechanism proposed in the previous section is integrated into the monitoring but, this time, is coupled to alarms that isolate faults present between the turbidity sensors. These instruments will provide continuity to the estimation process in the event of failures in one of them. Two turbidity sensors are used since, with the biomass concentration, the rest of the concentrations can be estimated, such as substrate, ethanol, and $CO_{2}$, for which no sensors are available. Therefore, it is necessary to guarantee that this measurement is correct since the monitoring of the rest of the state variables depends on it.

**Figure A1:**
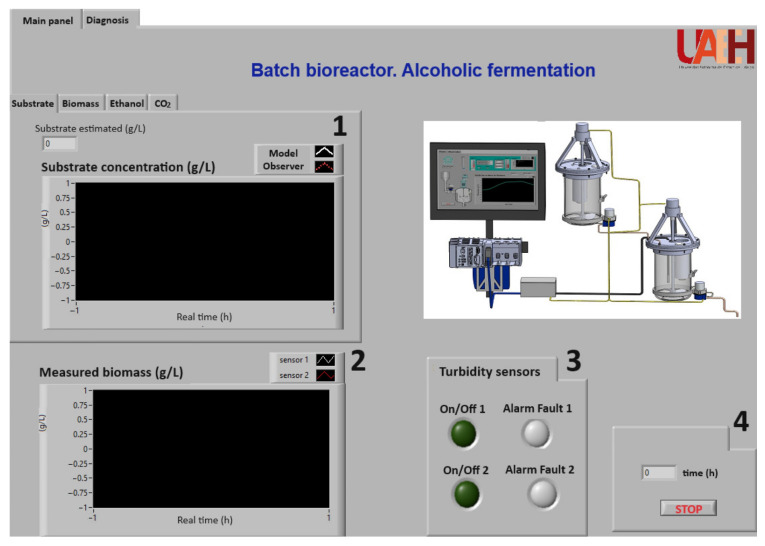
Main panel distribution of the HMI interface. 1—Estimated variables. 2—Measured output. 3—Alarm faults. 4—Operation time and stop button.

**Figure A2:**
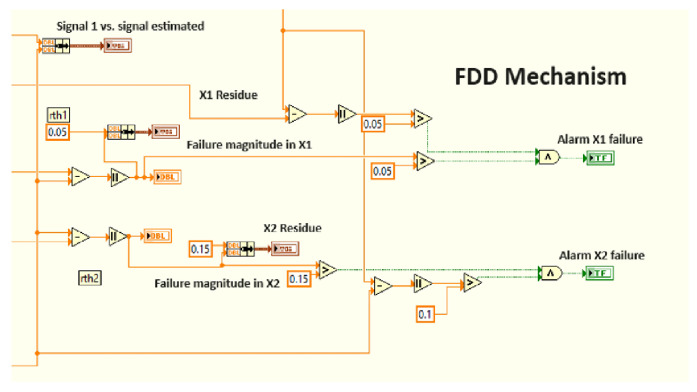
FDD mechanism monitoring.

For this reason, the biomass signals will be the inputs for each observer of the diagnostic bank since, if a malfunction occurs in one of them, the other guarantees the continuity of the process and the user can be alerted in a timely manner (see Appendix A). The HMI interface is distributed in two main panels (see Figure A1 and Figure A2).

Initially, residual error is defined as the absolute difference between the process result and the process models during normal operation and in the event of failure. Next, an acceptable error threshold is determined based on historical data and heuristic knowledge of the process to indicate that the process is fault-free. Fault detection and diagnosis are carried out through initial real-time verification, followed by fault detection performed by measuring residual values. To avoid this problem that can have serious consequences if the correct monitoring information is not provided, an FDD mechanism based on diagnostic observers and a dedicated observers scheme (DOS) is proposed.

To achieve the desired result of detecting faults in the sensor, we suggest a special kind of evaluation function. This is based on the root-mean-square (RMS) value of the residual and a practical threshold for the bioreactor. Sensor fault detection is achieved by considering a residual evaluation function, which is given by

(1) $$\delta_{RMS}=∥r_{i_{RMS}}∥=\delta \left(\sigma ,t\right).$$

Here,

(2) $$\delta \left(\sigma ,t\right)=\sqrt{\frac{1}{\Delta t}\int_{t-\Delta t}^{t}r^{T}\left(\sigma \right)r\left(\sigma \right)d\sigma }.$$

The RMS value measures the average of the residual signal $r_{i}\left(t\right)$, throughout the stipulated time period [t−Δ,t] based on [24].

## 3. Mathematical Model for Bioreactor Operating in Batch

Experimental validation for the mathematical model.

The strain used was *Pichia anomala*, CDBB 1446, ATCC 8168, CINVESTAV, which was inoculated in a 250 mL flask with 150 mL of sterile medium, incubated at 33 °C and 250 rpm with 15 (*v*/*v*) of *Pichia anomala*. *Pichia anomala* carries out alcoholic fermentation, converting glucose into ethanol and $CO_{2}$ in an anaerobic environment while consuming glucose to produce biomass. This yeast strain is used in biofuel production, and studies focus on optimizing fermentation by controlling oxygen availability to favor ethanol production over other metabolic pathways. *P. anomala* was selected as the biocatalyst due to its proven ability to produce bioethanol from various types of food waste and residues, with ethanol yields reaching up to 85% of the theoretical maximum. For strain reactivation and fermentation, a modified minimal synthetic medium was used and adjusted: glucose, 20 g/L; $MgSO_{4}$·$7H_{2}O$, 0.4 g/L, $KH_{2}PO_{4}$, 5 g/L; $\left(NH_{4}\right)2SO_{4}$, 2 g/L; yeast extract, 1 g/L, pH 5.5.

In the experimental stage, the juices extracted from the cocoa pulp were adjusted to 20 g/L of glucose using reducing sugars. Every 5 h for 60 h, 5 mL samples were taken. The cell count was determined by dry weight. In addition, the dinitrosalicylic acid (DNS) colorimetric method was used to estimate total reducing sugars. The samples were centrifuged (15,000 rpm, 10 min), cooled (5 °C), and filtered (0.22 µm) before being injected into the high performance liquid chromatograph (HPLC).

The growth kinetics of the microorganism is then described when the bioreactor is operated in batch using a system of differential equations. In this case, loading is performed only once at the beginning of the process, and the product is not unloaded until after the fermentation has concluded (retention time).

Mathematical model

*Substrate balance:*

(3) $$\dot{S}=-\kappa_{1}\left(S^{\kappa_{5}}X^{\kappa_{6}}\right)$$

*Biomass:*

(4) $$\dot{X}=\kappa_{2}\left(S^{\kappa_{5}}X^{\kappa_{6}}\right)-\kappa_{11}Et^{\kappa_{7}}CO_{2}^{\kappa_{8}}$$

*Ethanol balance:*

(5) $$\dot{Et}=\kappa_{3}\left(S^{\kappa_{5}}X^{\kappa_{6}}\right)-\kappa_{10}\left(1-\frac{Et}{\kappa_{9}}\right)$$

*$CO_{2}$ balance:*

(6) $$\dot{CO_{2}}=\kappa_{4}\left(S^{\kappa_{5}}X^{\kappa_{6}}\right)CO_{2}^{\kappa_{7}}$$

In the same way, we have to consider the following:

- $\kappa_{1}$, $\kappa_{2}$, $\kappa_{3}$, and $\kappa_{4}$ are the specific velocities for the concentrations of S,X,Et, and $CO_{2}$, respectively.
- $\kappa_{5}$, $\kappa_{6}$, $\kappa_{7}$, and $\kappa_{8}$ are exponential terms that constitute the order of the reaction with respect to the reactant to which a particular concentration is elevated.
- $\kappa_{9}$ is the inhibition factor of ethanol.
- $\kappa_{10}$ is the inhibition constant of the enzymatic activity.
- $\kappa_{11}$ is the inhibition constant of biomass with respect to ethanol.

The values of the process parameters used are reflected in Table 2.

**Figure 3:**
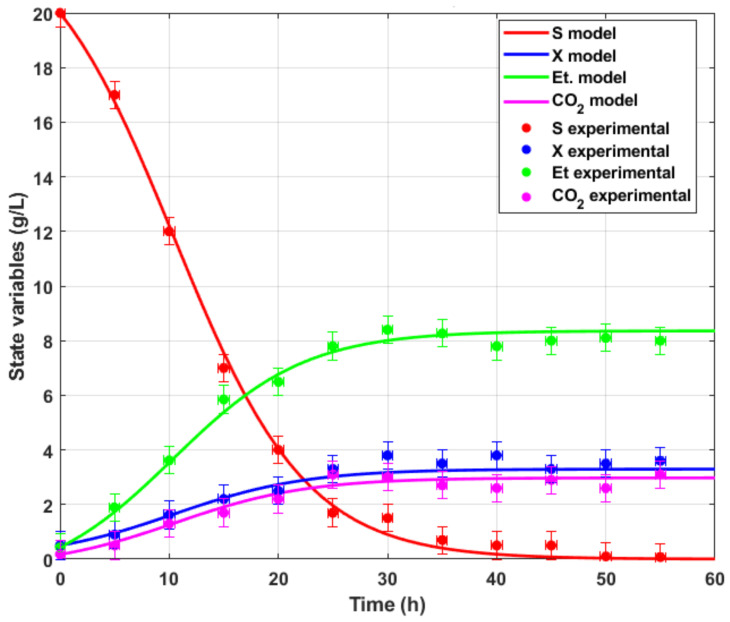
The validation of the model is based on a comparison of simulation data with experimental data.

Figure 3 shows the experimental data of the bioreactor operating in batch and the corresponding variables of the mathematical model (3)–(6). The concentration of $CO_{2}$ was determined with the $CO_{2}$-BTA Vernier probe; therefore, the production of $CO_{2}$ is monitored because the yeast consumes different initial glucose concentrations. The optimized parameters obtained by the Levenberg–Marquardt algorithm method agree well with the experimental data compared with the simulations. The predictions of the model using experimental observations provided acceptable performance values averaging $R^{2}=0.946$.

### 3.1. Model Linearization

The form of the nonlinear system given by [25]

$$\begin{array}{cc} \dot{x}= & f(x,u)\\ y= & h(x) \end{array}$$

where $x\in R^{n}$ is a vector of state variables, u∈R is a scalar input, y∈R is a scalar output, $f:R^{n}\times R\to R$ is a nonlinear vector function in terms of the state and input variables, and $h:R^{n}\to R$ is a scalar function that depends on the state vector. Next, it is evaluated at the equilibrium points of the system to obtain the linearized system, and in this way, the state matrix *A* that represents the process is obtained. On the other hand, the output matrix *C* is found, taking into account the variable that is measured online: biomass.

Developing the gradient of *f* for each state variable, we are left with the dimension of *A* being 4×4.

(7) $$A=\nabla_{\times }f{|}_{x=\bar{x}}$$

To calculate the matrix *A*, use the jacobian command in Matlab^®^ and subsequently evaluate the result at each operating point, obtaining

(8) $$A=\left[\begin{array}{cccc} 1241 & -0.0064 & 0 & 0\\ 0.0229 & -0.0001 & -8.6426\times 10^{8} & -2.4260\times 10^{-8}\\ 0.0035 & 0.0001 & -0.001 & 0\\ 0.0231 & 0.0009 & 0 & -0.001 \end{array}\right]$$

The linearized system for batch operation has the form

(9) $$\begin{array}{cc} \dot{x}= & Ax+Bu\\ y= & Cx \end{array}$$

In terms of control input, the system is considered autonomous during the reaction phase (when B=0 or *u* is a null vector), since the dynamics are driven by initial conditions and inherent biological kinetics rather than by external mass flows. *B* is defined in such a way that it directly maps the control variables to the corresponding state. If there is no external control variable (such as a feed flow), then *u* can be treated as a disturbance vector, or the system can simply be treated as an autonomous system ($\dot{x}=f\left(x\right)$).

The output vector *C* considers the online measurement of the biomass variable, and from this measurement, the entire state vector can be estimated; therefore, *C* is chosen in the form

$$C=\left[\begin{array}{cccc} 0 & 1 & 0 & 0 \end{array}\right]$$

### 3.2. Observability Analysis

**Theorem** **1**  (Kalman’s observability theorem [25]). *Given m, n ∈N, A $\in {ℜ}^{n\times n}$, C $\in {ℜ}^{m\times n}$, t $\in {ℜ}^{+}$, the system is considered*

(10) $$\begin{array}{cc} \dot{x} & =Ax+Bu\\ y & =Cx \end{array}$$

*Then, the system is said to be completely observable if there exists a signal u(t) that allows the initial states of the system $x_{0}=x\left(t_{0}\right)$ to be transferred to any other state $x_{tf}$ in a finite time $T=t_{f}-t_{0}$ [26].*

The definition of Kalman observability is applied to a system that runs in batches and continuously. The observability matrix **$Q_{0}$** is found, which must present a full range (necessary condition). In this way, the complete observability of the system can be guaranteed [27].

The construction of the observability matrix is carried out as follows [25]:

(11) $$Q_{0}=\left[\begin{array}{c} C\\ CA\\ CA^{2}\\ \vdots \\ CA^{n-1} \end{array}\right]$$

The rank of the observability matrix is 4. Therefore, the condition of complete observability of the linearized system is satisfied with the biomass concentration as input to the observer [26].

Table 3 shows the different configurations of the matrix *C* along with the state variables that can be estimated, represented by (•), while those that cannot be estimated (∘).

## 4. Robust Full-Order Adaptive Observer

### 4.1. Robust Full-Order Adaptive Observer Application Simulation

In this section, it is required to test the adaptive robust observer obtained under the mathematical formalism proposed above. To do this, the observed response implemented on the proposed mathematical model that describes the dynamics of the bioreactor is simulated. Using the LMI resolution method of Sedumi, and after making an adjustment to achieve a good tuning of the profit matrix, the system operating in batches is simulated with the concentration of simulated biomass. Table 4 shows some simulation characteristics in the adaptive observer application.

The observer tuning strategy employs a two-stage approach. First, stability conditions are resolved using an LMI to define the convergence region. Second, the adaptive parameters are adjusted to optimize the balance between response speed and noise rejection. The system’s robustness is bounded by the parameter δ (see Equation (13)), which defines the maximum level of uncertainty and noise that the observer can absorb before the RMS residual exceeds the detection threshold. Practical limitations to generalization include ensuring the structural observability of the new bioprocess and having a kinetic model that captures essential nonlinear dynamics available. The strategy is less effective in processes where the dynamics are dominated by unmodeled stochastic phenomena.

**Figure 4:**
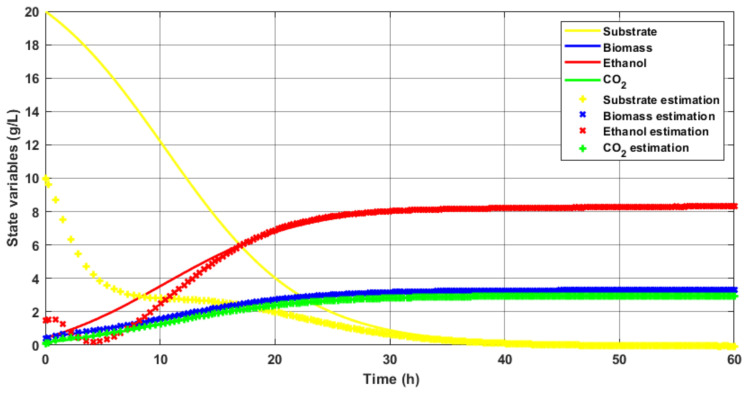
State variables estimation with robust full-order adaptive observer.

Figure 4 shows the result of the adaptive observer estimation in simulation for each state variable separately. In these cases, initial conditions for the observer are taken that are close to those of the process depending on the variable involved.

We can observe that, despite the different initial conditions in the substrate estimation with respect to the behavior of this variable, the oscillations in the transient state were slight, the overshoot was eradicated, and the convergence of both signals was guaranteed. For ethanol, the desired behavior was also achieved, as the oscillations were minimal, caused by the initial conditions and the dependence of the ethanol on the substrate. The biomass and $CO_{2}$ signals performed correctly in the process in both the transient and steady states.

As was previously reflected, the adaptive observer had a good estimation response in the transient part, achieving damping of the oscillations and without showing overshoot peaks. On the other hand, it achieved a compensation in convergence to zero of the steady-state error.

To check the performance of the adaptive robust observer in terms of robustness and adaptability, the proposed model was subjected to a set of unfavorable conditions that could be present within an industrial environment in real time.

Drastic changes to the model parameters were made (see Table 5), simulating parametric uncertainties. In addition, white noise was added to the biomass balance, simulating the noise at the output of the turbidity sensor that measures biomass. To test the law of observer adaptability, the initial conditions of the observer were altered and the model’s parameter values were verified to determine whether the observer could truly follow the trajectory (see Figure 4). The observer gain matrix was modified under these unfavorable conditions to which the plant was subjected, obtaining the values *ℓ* =[−30;−20;−380;−2.1].

### 4.2. Robust Observers

The design of the observers is based on the nominal dynamics defined in the system (9). This model incorporates both uncertain dynamics, derived from higher-order terms that were omitted during linearization, and parametric uncertainties. Based on these premises, the following dynamic structure is established:

(12) $$\begin{array}{c} \dot{x}=Ax+Bu+\xi \\ y=Cx\; \end{array}$$

where ξ represents the parametric uncertainties and unmodeled dynamics, under arbitrary initial conditions $x\left(0\right)=x_{0}$. It is assumed that ξ satisfies the bounded condition

(13) $${\left||\xi |\right|}^{2}\leq \delta .$$

In this case, consider that the pair (A,C) is observable; then a full-order observer with adaptive gain is proposed, with the following structure:

(14) $$\dot{\hat{x}}=A\hat{x}+Bu-\ell \left(y-C\hat{x}\right)\;$$

where *ℓ* $\in {ℜ}^{n}$ is the adaptive observer gain, which will be designed in Proposition 1.

Let us define the observation error as

(15) $$\zeta =x-\hat{x}\;$$

Taking the time derivative of Equation (15), and substituting Equations (12) and (14),

(16) $$\dot{\zeta }=\left(A+\ell C\right)\zeta +\xi \;$$

The following proposition defines conditions to guarantee that the observer (14) will be functional.

**Proposition** **1**  (Full-Order Adaptive Robust Observer). *Consider that the observation gain matrix has the following adaptation rule:*

(17) $$\dot{\ell }\left(t\right)=-\frac{\gamma }{2}\tilde{\ell }-\frac{1}{\gamma }P\Lambda^{-1}P\tilde{\ell }\left(y-C\hat{x}\right)\left(y^{T}-{\hat{x}}^{T}C^{T}\right)$$

If there is a set of solutions for $0<P\in {ℜ}^{n\times n}$, $0<\Lambda =\Lambda^{T}\in {ℜ}^{n\times n}$, $\tilde{\ell }=\ell \left(t\right)-\ell^{*}$, and $\ell \in {ℜ}^{n}$ that satisfies the following matrix inequality:

(18) $$W=\left[\begin{array}{cc} \left(A^{T}+C^{T}\ell^{*T}\right)P+P\left(A+\ell^{*}C\right)+\alpha P+\Lambda & P\\ P & -\varepsilon I \end{array}\right]<0$$

*where $\alpha \in {ℜ}^{+}$, $\varepsilon \in {ℜ}^{+}$, then the full-order adaptive robust observer* (14) *estimates the state variables of system* (12)*. Additionally, it is guaranteed that the estimation error* (15) *is uniformly ultimately bounded stable.*

**Definition** **1**  (Uniformly ultimately bounded (UUB) stability). *The solution of system* (16) *is said to be UUB stable if it is uniformly stable and there exists d>0 with ultimate bound h>0 independent of $t_{0}$, that is, for k∈(0,d) exists Γ=Γ(k,h)>0, such that if $∥\zeta (t_{0})∥\leq k$, then $∥\zeta (t)∥\leq h,$ for all $t\geq t_{0}+\Gamma$.*

**Lemma** **1.**  *Let the scalar function V(t) that satisfies the following inequality:*

(19) $$\frac{d}{dt}V\left(t\right)+\alpha V\left(t\right)\leq \beta$$

*With $V\left(t_{0}\right)=V_{0}$. Then, for all $\alpha \in {ℜ}^{+}$ and $\beta \in {ℜ}^{+}$, the function V(t) is UUB stable, with*

(20) $$h=\frac{\beta +\alpha \gamma }{\alpha },\;\Gamma =\frac{1}{\alpha }ln\left\{\frac{\alpha V(t_{0})-\beta }{\beta \gamma }\right\}+t_{0}$$

*for a small enough $\gamma \in {ℜ}^{+}$.*

**Proof of the Proposition** **1.**  Let us consider the energy function

(21) $$V\left(\zeta ,\tilde{\ell }\right)=\zeta^{T}P\zeta +\frac{\gamma }{2}tr\left[{\tilde{\ell }}^{T},\ell \right]$$

where $P=P^{T}>0$, γ>0, and $\tilde{\ell }=\ell \left(t\right)-\ell^{*}$. Taking into account the properties of the trace function yields the following:

(22) $$\frac{\gamma }{2}tr\left[{\tilde{\ell }}^{T},\dot{\tilde{\ell }}\right]+\frac{\gamma }{2}tr\left[{\dot{\tilde{\ell }}}^{T},\tilde{\ell }\right]=\gamma tr\left[{\tilde{\ell }}^{T},\dot{\tilde{\ell }}\right]$$

The time derivative of Equation (21) along the trajectories of Equations (16) and (22) is expressed as

(23) $$\begin{array}{c} \dot{V}=\zeta^{T}\left[\left(A^{T}+C^{T}\ell^{T}\left(t\right)\right)P+P\left(A+\ell \left(t\right)\right)C\right]\zeta +\xi^{T}P\zeta +\zeta^{T}P\xi +\gamma tr\left[{\tilde{\ell }}^{T},\dot{\ell }\left(t\right)\right] \end{array}$$

Since $\tilde{\ell }=\ell \left(t\right)-\ell^{*}$, then its time derivative is given by $\dot{\tilde{\ell }}=\dot{\ell }\left(t\right)$, and by the addition and subtraction of $\zeta^{T}\left[C^{T}\ell^{*T}P+P\ell^{*}C\right]\zeta$, Equation (23) is equivalent to

(24) $$\begin{array}{c} \dot{V}=\zeta^{T}\left[\left(A^{T}+C^{T}\ell^{*T}\right)P+P\left(A+\ell^{*}C\right)+\alpha P\right]\zeta +\xi^{T}P\zeta +\zeta^{T}P\xi +\gamma tr\left[{\tilde{\ell }}^{T},\dot{\ell }\left(t\right)\right]\\ \;\;\;\;\;\;\;\;\;\;\;\;\;\;\;\;+\alpha \frac{\gamma }{2}tr\left[{\tilde{\ell }}^{T},\tilde{\ell }\right]-\alpha +{\varepsilon ||\xi ||}^{2}-{\varepsilon ||\xi ||}^{2}+\zeta^{T}\left[C^{T}{\tilde{\ell }}^{T}P+P\tilde{\ell }C\right]\zeta \end{array}$$

Let us define the augmented vector ${\left[\zeta \;\xi \right]}^{T}$; Equation (24) can be written as

(25) $$\begin{array}{c} \dot{V}=\left[\zeta \;\xi \right]\left[\begin{array}{cc} \left(A^{T}+C^{T}\ell^{*T}\right)P+P\left(A+\ell^{*}C\right)+\alpha P & P\\ P & -\varepsilon I \end{array}\right]\left[\begin{array}{c} \zeta \\ \xi \end{array}\right]-\alpha +{\varepsilon ||\xi ||}^{2}\\ \;\;\;\;\;\;\;\;\;\;\;\;\;\;\;\;\;\;\;\;\;+tr\left[{\tilde{\ell }}^{T}\left(\gamma \dot{\ell }\left(t\right)+\frac{\alpha \gamma }{2}\tilde{\ell }\right)\right]+\zeta^{T}\left[C^{T}{\tilde{\ell }}^{T}P+P\tilde{\ell }C\right]\zeta \end{array}$$

The last term in Equation (25), $\zeta^{T}\left[C^{T}{\tilde{\ell }}^{T}P+P\tilde{\ell }C\right]\zeta =\zeta^{T}C^{T}{\tilde{\ell }}^{T}P\zeta +\zeta^{T}P\tilde{\ell }C\zeta$, is simplified as

(26) $$\zeta^{T}C^{T}{\tilde{\ell }}^{T}P\zeta +\zeta^{T}P\tilde{\ell }C\zeta =2\zeta^{T}P\tilde{\ell }C\zeta$$

By using Equations (12) and (15), we have

(27) $$P\tilde{\ell }C\zeta =P\tilde{\ell }\left(y-C\hat{x}\right)$$

Then, we get the following:

(28) $$\begin{array}{c} \dot{V}\leq \left[\zeta \;\xi \right]\left[\begin{array}{cc} \left(A^{T}+C^{T}\ell^{*T}\right)P+P\left(A+\ell^{*}C\right)+\alpha P+\Lambda & P\\ P & -\varepsilon I \end{array}\right]\left[\begin{array}{c} \zeta \\ \xi \end{array}\right]\\ \;\;\;\;\;\;\;\;-\alpha +{\varepsilon ∥\xi ∥}^{2}+tr\left[{\tilde{\ell }}^{T}\left(\gamma \dot{\ell }\left(t\right)+\frac{\alpha \gamma }{2}\tilde{\ell }\right)+P\Lambda^{-1}P\tilde{\ell }\left(y-C\hat{x}\right)\left(y^{T}-{\hat{x}}^{T}C^{T}\right)\right] \end{array}$$

By considering (17) and (18), the following upper bound $\dot{V}\left(\zeta ,\tilde{\ell }\right)$ is verified:

(29) $$\dot{V}\left(\zeta ,\tilde{\ell }\right)\leq -\alpha V+\varepsilon \delta$$

Then, by Lemma 1, *V* is UUB stable.    □

The proofs of the stability, robustness, and adaptability of *W* are presented and detailed in [26].

## 5. Fault Detection and Diagnosis Mechanism

For the development of this module, the essence of some detection and diagnosis methods is used, such as model-based with diagnostic observers (residual generation method), analytical redundancy, and other model-free methods, such as physical redundancy and signal analysis [27]. To test the proposed algorithm, the procedures for the bioreactor to operate in batch are initially simulated. Sensor failures are taken into account, ignoring those that may occur in actuators, since for this type of operation, there are no input or output flows [28]. To test the simulation, it is necessary to detect and isolate faults in the turbidity sensor and the $CO_{2}$ probe using the method based on state observers. To do this, we rely on the dedicated observer scheme (DOS), which has the advantage of detecting and isolating faults that occur simultaneously using banks of observers and estimation residuals as a technique [29].

**Figure 5:**
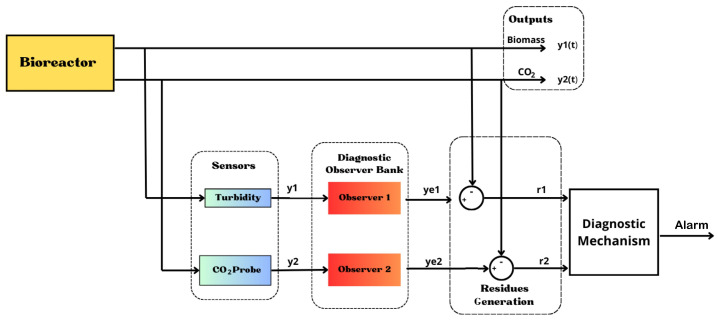
Sensor fault detection and diagnosis module based on DOS scheme.

The proposed scheme is based on the fact that each bank observer will receive only the signal from a measuring instrument at their entrance. With each of them, the observability of the states measured online must be guaranteed [30]. Subsequently, each observer will generate a residual to compare with a reference threshold that will trigger certain alarms when the detection thresholds are exceeded [31]. Taking this observation into account and following the classical residual-based FDI schemes, the detection of a failure follows the following logic:

- $fault_{i}=\mathrm{true}$, si $r_{i}\left(t\right)>r_{i,\mathrm{th}}$,
- $fault_{i}=\mathrm{false}$, si $r_{i}\left(t\right)\leq r_{i,\mathrm{th}}$,

Where *i* is the number of sensors to detect failures, where in this case there will be two, the turbidity sensor for biomass and the Vernier $CO_{2}$ probe, and $r_{i}$(t) are the residuals generated by each sensor, taking into account that $r_{i}\left(t\right)=\hat{y}-y$. Furthermore, $r_{ith}$ are fixed detection thresholds (*thresholds*) for each output [32]. The estimation results can be improved, and in this way, an FDD module is obtained that provides complete information on the occurrence, location, and magnitude of the failure that occurred [33]. To guarantee FDD in the sensors, three possible scenarios are tested in real time: isolated failure in turbidity sensor 1, isolated failure in the Vernier probe $CO_{2}$, and the occurrence of simultaneous failures in both sensors. However, the biomass estimation results are only presented to validate the performance of the estimator with respect to the real-time sensor signal. Each dedicated observer uses measurements from a specific sensor to generate a residual sensitive to faults in that sensor, but largely unaffected by faults in other sensors. The system can detect, locate, and isolate single or multiple simultaneous sensor faults by comparing these unique residuals with thresholds, ensuring system reliability and stability. Additionally, Figure 5 provides two outputs from the bioreactor, which are fed individually to the DOS algorithm, as well as the dynamics generated by the model-based observer, which are implemented independently for each sensor.

**Figure 6:**
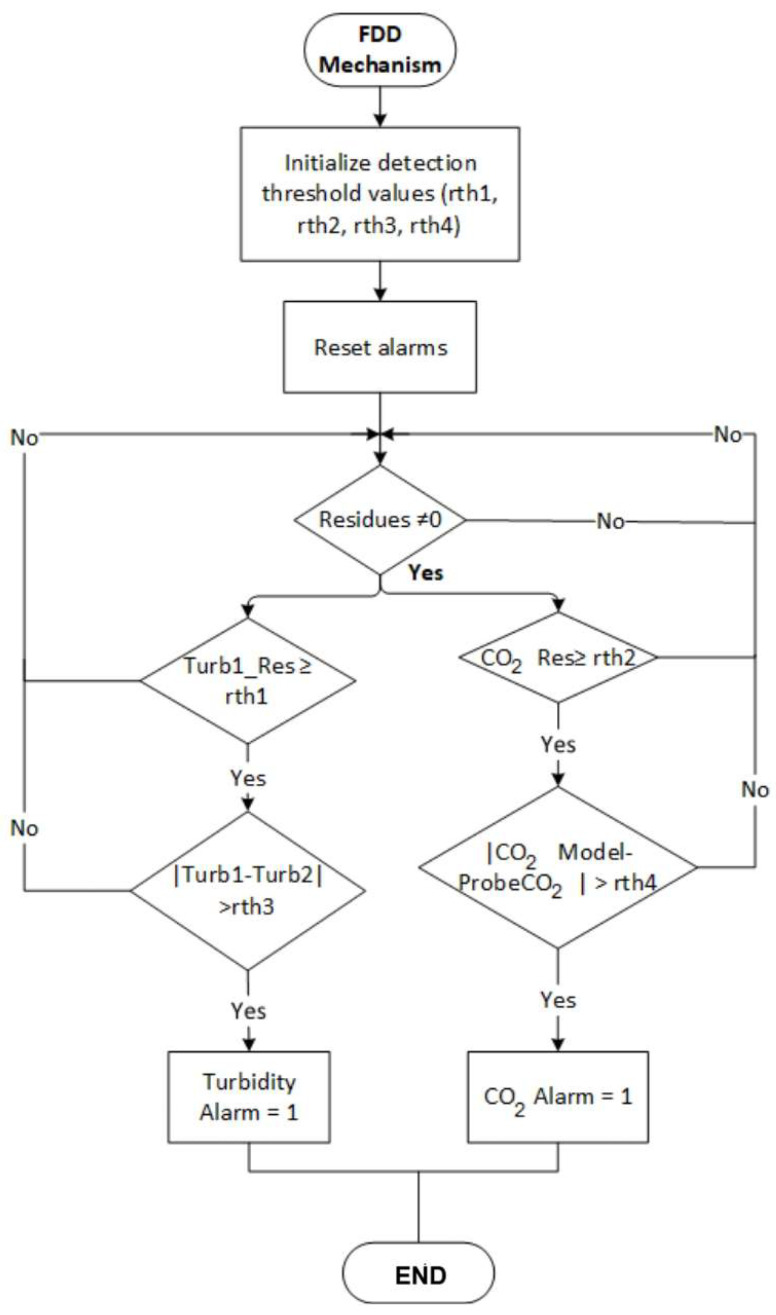
Fault detection and diagnosis algorithm.

The system is developed as shown in Figure 5; a DOS scheme is used, where the output of turbidity sensor 1 is received at the input of observer 1; and the received signal is received at observer 2. of the $CO_{2}$ probe. The first bench observer is the robust full-order adaptive one that was developed earlier. In addition, there is a second turbidity sensor located as physical redundancy; in case failures occur in the first, it is replaced by the second, and thus the process continues to estimate the state variables, providing continuity to the process [34]. Next, in Figure 6, the diagnostic algorithm is proposed, which represents in a flow chart the programming logic for the evaluation of residues.

For this observer 1, the signal from turbidity sensor 1 is taken as input, which represents the biomass concentration in the reactor. The measured variable (biomass) guarantees complete observability of the states. Therefore, if a failure occurs in the $CO_{2}$ probe, since observer 1 does not have this input, the estimation of all state variables will be based on a correct measurement. This technique proposed by the DOS mechanism will serve as a way to detect and isolate faults in the $CO_{2}$ probe. On the other hand, for the second observer of the bank, also a full-order robust adaptive, the signal delivered by the $CO_{2}$ probe is required as input to the observer, which guarantees the observability of the concentrations of substrate, biomass, and $CO_{2}$. In this way, if a failure occurs in turbidity sensor 1, observer 2 will not estimate it, since, at its input, it does not have this failed measurement.

Therefore, this mechanism will be viable to detect and diagnose failures in turbidity sensor 1. Given the simultaneous occurrence of failures in both sensors or in each one separately at different times of the process, a comparison is made with the experimentally validated mathematical model that faithfully represents the dynamics that the system must follow for the concentration signals, biomass and $CO_{2}$.

In Matlab^®^’s Simulink platform, the signals are acquired in real time and are associated with the FDD mechanism programmed to diagnose failures in the event of malfunction of both sensors. The generation of waste and the behavior of faulty signals are visualized using oscilloscopes located within the program. In the same way, the FDD mechanism is programmed to analyze the operation of the measuring instruments during the fermentation process and trigger alarms in the event of their malfunction, as shown in Figure 6. Next, Table 6 is proposed, which shows the value of the profit matrices of the process observers and those that make up the diagnostic bank. In addition, the initial conditions of the observers used are shown. These outputs constitute the inputs for each bank observer, which generate residuals based on the failures that occur.

### 5.1. Case 1: Abrupt Failure in Turbidity Sensor *1*

**Figure 7:**
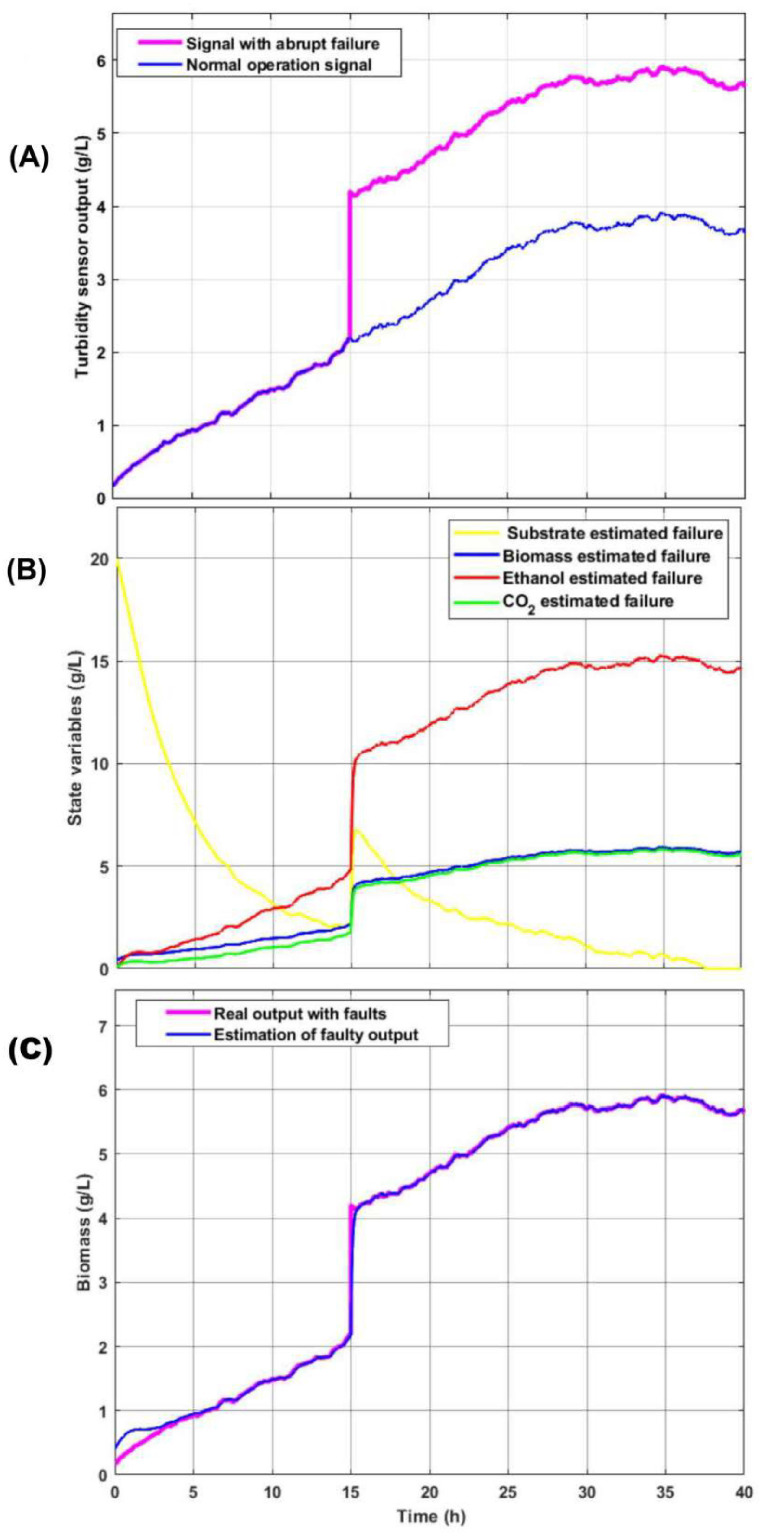
Analysis of abrupt sensor failure during the bioprocess (**A**) Comparison between the turbidity sensor output under normal operation (blue) and with an abrupt failure (magenta) at t=15 h. (**B**) Dynamics of the estimated state variables (substrate, biomass, ethanol, and $CO_{2}$) showing the fault propagation. (**C**) Performance of the fault estimation and reconstruction, comparing the real faulty output against the estimated signal.

As presented in Figure 7A, at 15 h, the signal from turbidity sensor 1 is disturbed with an amplitude step of 2 g/L, as seen. Below, the output of the observers from the implemented bank is illustrated, the first estimating the biomass signal with an abrupt failure at 15 h and the second correctly estimating the biomass concentration since it is not presented as signal turbidity sensor 1 faulty.

In the event of sensor failure, the action of the observer bank intervenes, leaving the signal in Figure 7B as the estimated failed signal in the output of observer 1. Let us remember that the first observer of the bench has, at its entrance, the output of the turbidity sensor that guarantees the estimation of the state variables for monitoring the process. Below is the behavior of the state variables estimated by the first observer of the bank that is estimated through the output of the failed turbidity sensor 1. As seen in Figure 7C, there is a considerable residual in the observed variables. To restore its correct condition, the failed turbidity sensor 1 must be replaced in the shortest possible time, since the correct estimation of the variables that cannot be measured online depends on it: substrate concentration, ethanol, and $CO_{2}$. The following is the erroneous estimation of the state variables when a failure occurs in turbidity sensor 1. This results in the incorrect monitoring of the estimated variables not being achieved.

**Figure 8:**
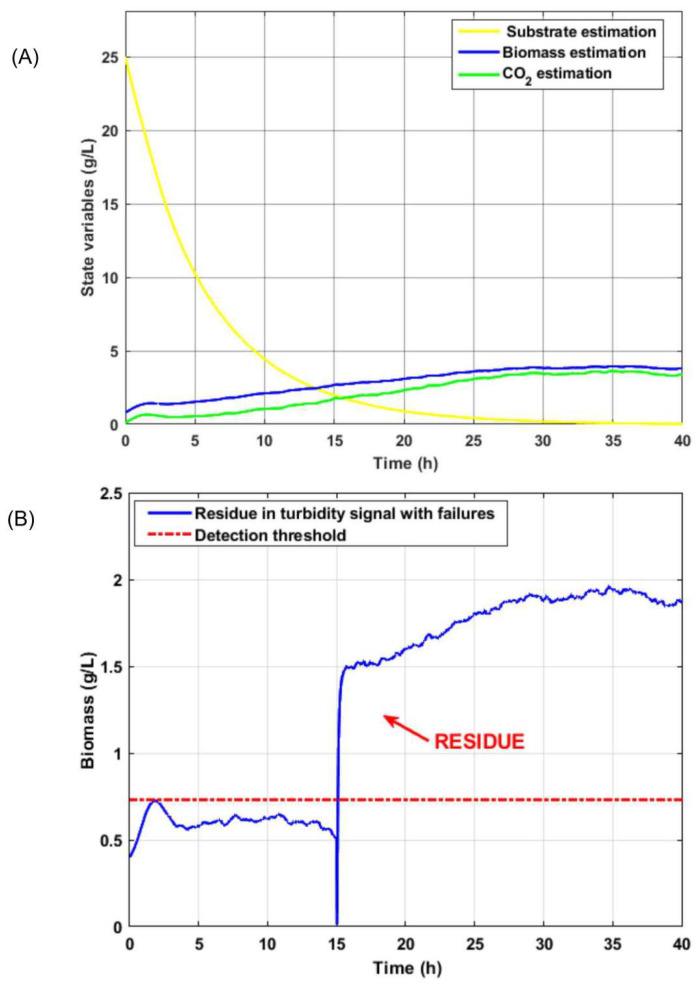
State estimation and fault detection performance. (**A**) Evolution of the estimated state variables (substrate, biomass, and CO_2_) under normal operating conditions. (**B**) Residual signal generated from the turbidity sensor; the red dashed line indicates the detection threshold, showing a clear crossing at t=15 h due to the sensor failure.

It is interesting to show in the case of the second observer of the bank that, by not counting the failed signal from turbidity sensor 1, the correct estimation of the three observable state variables for this output is reached: substrate, biomass, and $CO_{2}$ (see Figure 8A. Since biomass is an observable state with the concentration of $CO_{2}$ as output, it is valid to make a comparison between the output of the second observer of the fault-free bank with respect to the output of the first observer with faults. With this procedure, a residue is generated that indicates the occurrence of an anomaly on the first sensor. In Figure 8B, a residue is observed after 15 h, which is when an abrupt failure occurs in turbidity sensor 1. The signal is not restored since the replacement of the turbidity sensor was not carried out, turbidity sensor 1 for the remaining time of the process.

### 5.2. Case 2: Abrupt Failure in Probe $CO_{2}$

**Figure 9:**
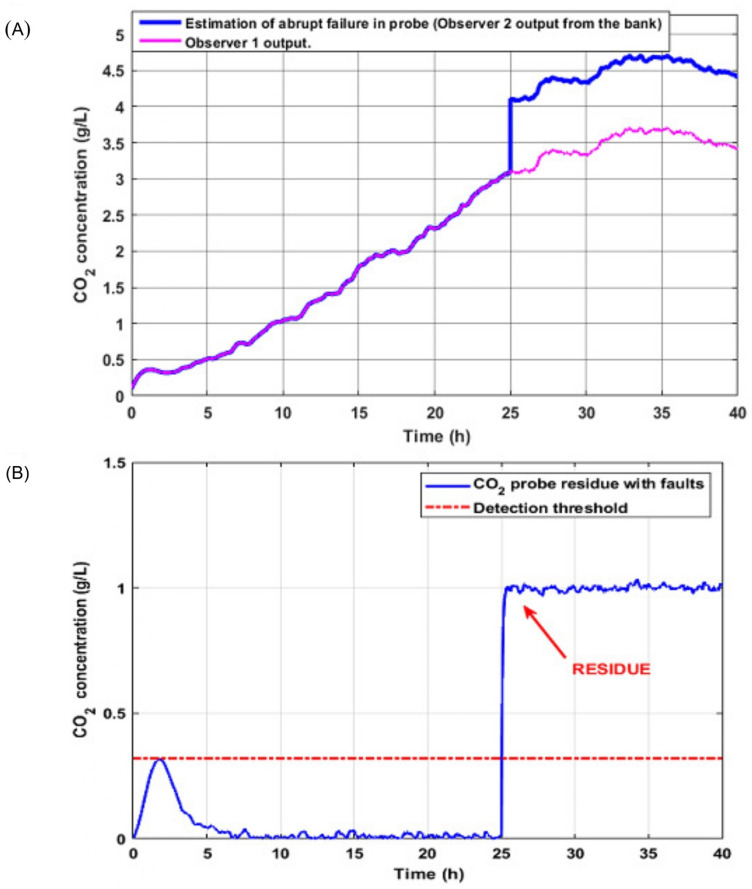
(**A**) Signal from the $CO_{2}$ probe with abrupt failure vs. correct operation; (**B**) residue generated due to abrupt failure of the $CO_{2}$ probe.

Subsequently, it was tested that the mechanism worked in real time for abrupt failure in the $CO_{2}$ probe at 25 h and with an amplitude of 1 g/L (see Figure 9A). The following figure shows the output of each bank observer for fault diagnosis. In magenta, the output of observer 1 is represented, which is estimated with the output of the fault-free turbidity sensor. However, the blue line illustrates the behavior in the output of the second observer of the bank whose input belongs to the probe with abrupt failure.

Next, the behavior of the residual generated between the output of the first observer with a fault-free input and the second observer of the bank that does have the malfunctioning probe signal at its input is observed. Since it is an abrupt type of failure, detection occurs instantly; its behavior is abrupt and dangerous since it can lead the system to instability (see Figure 9B).

### 5.3. Case 3: Abrupt Failures with Simultaneous Occurrence in the Turbidity Sensor and Probe $CO_{2}$

Finally, the operation of the mechanism was tested in the event of sudden failures with simultaneous occurrence in both sensors 20 h after starting the process.

**Figure 10:**
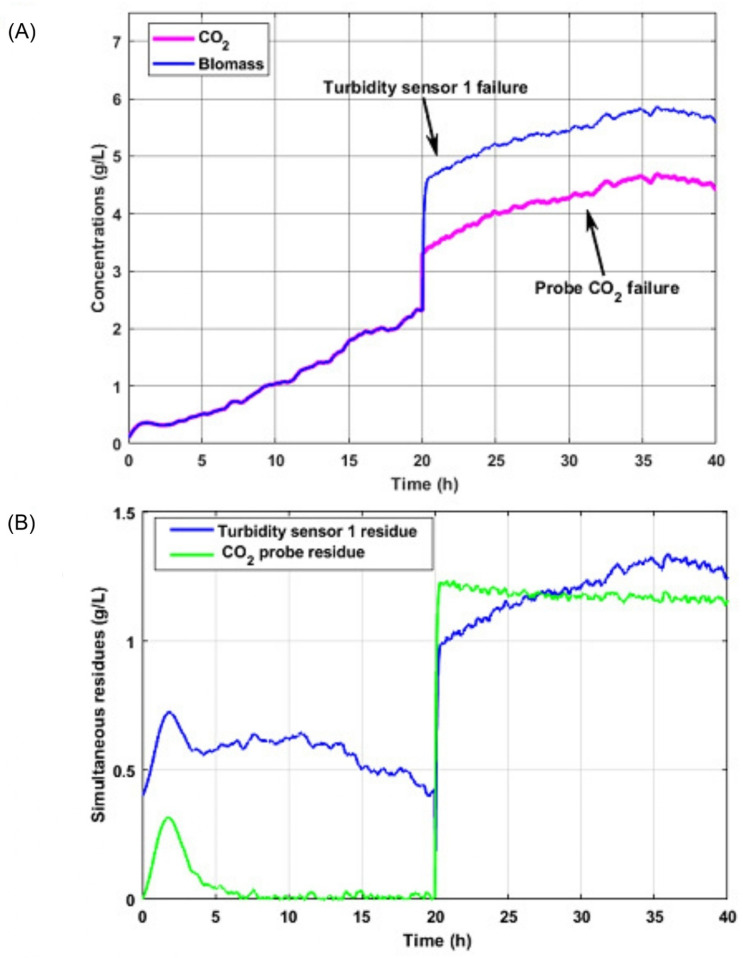
Estimated signals with abrupt simultaneous failures in turbidity sensor 1 and $CO_{2}$ probe. (**A**) Biomass and $CO_{2}$ concentrations; (**B**) turbidity sensor 1 and $CO_{2}$ residues.

Figure 10A shows the results of the biomass behavior and signals $CO_{2}$ in the event of simultaneous failures. These graphs constitute the output of both observers of the implemented bank, which is a reflection of the failed estimates for these state variables.

In Figure 10B, the residual generated between the first bank observer with inputs to the failed turbidity sensor 1 and the output of the second turbidity sensor for biomass is represented. In addition, the output of the second observer from the bank and the dynamics of the $CO_{2}$ concentration are shown according to the mathematical model for the said variable.

**Figure 11:**
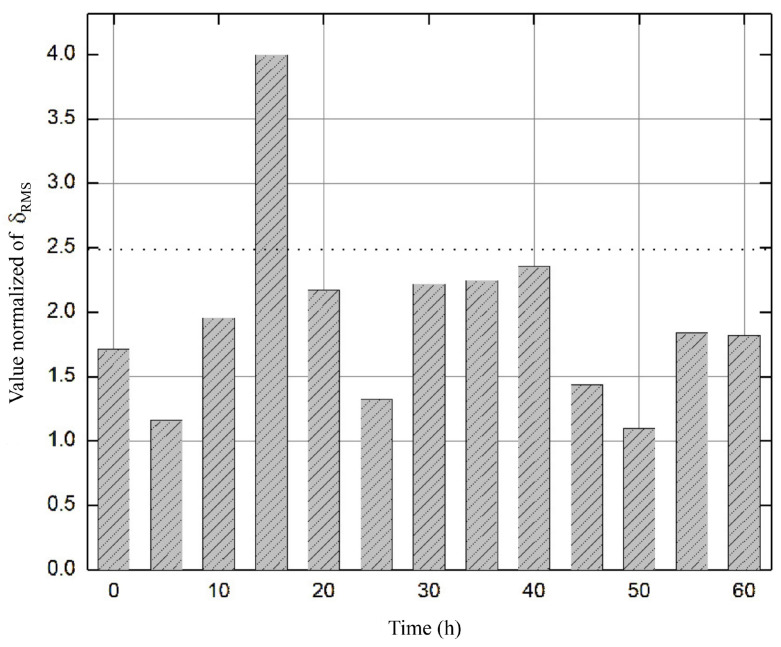
The FDD system corrects residual signals that are outside the threshold set under sensor fault conditions for case 1.

**Figure 12:**
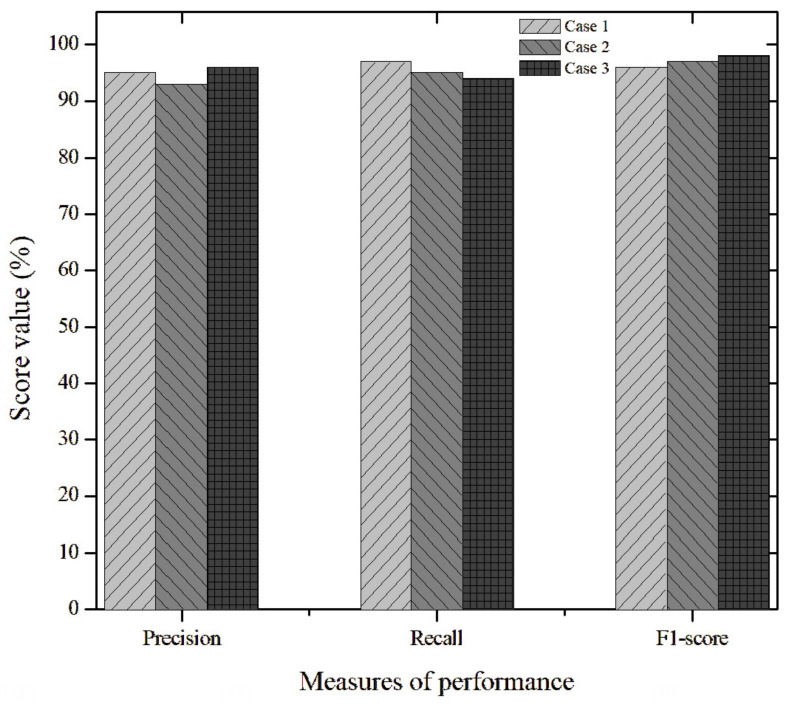
The performance measurements of algorithms in terms of F1-score, precision, and recall.

Thus, the benefits of the proposal in this contribution can be summarized in the following:

- A robustness analysis was performed to formally justify the detection thresholds, considering the upper bound of external disturbances δ defined in Equation (13). The RMS threshold was chosen to ensure that the detector is immune to variations within the set ξ and that $\dot{\zeta }$ remains within stable limits under nominal operation, according to the dynamics of Equation (16). The selection of the RMS threshold is not purely heuristic; it is based on the robustness property of the adaptive observer defined in Equations (14) and (17). Since the estimation error is bounded by the magnitude of the external disturbances, i.e., ${\left|\xi \right|}^{2}\leq \delta$, a sensitivity analysis was performed to determine the maximum expected residual under nominal conditions. The threshold $J_{th}$ was defined by a robustness analysis, ensuring that $J_{th}>\mathrm{RMS}\left(r_{nominal}\right)$ for all ξ that satisfy the bound δ. The threshold allows the detection of deviations greater than the jump observed in Figure 10 (turbidity sensor output) after *t* = 15 h. Finally, a robustness analysis based on observer error dynamics was performed to overcome the heuristic selection of detection thresholds. Assuming that external disturbances are bounded by ${\left|\xi \right|}^{2}\leq \delta$, the residual estimation error under nominal conditions remains within a compact invariant set. The threshold $J_{th}$ is formally defined by calculating the maximum expected steady-state root-mean-square (RMS) value for the fault-free system, as follows:
  $$J_{th}=\underset{t\in [t_{ss},T]}{\sup}\mathrm{RMS}\left(r\left(t\right)\right)+\sigma ,$$
  where $r\left(t\right)=y\left(t\right)-C\hat{x}\left(t\right)$ is the residual and σ is a confidence margin that compensates for the variance in the measurement noise observed in the experimental data.
- The proposed scheme is superior since it eliminates the computational training time required by methods such as in [35], which rely on hyperparameter optimization and large databases to achieve acceptable diagnostic accuracy. Although hybrid strategies [36,37] can exhibit detection delays due to signal processing and model complexity, our results show virtually instantaneous fault detection at *t* = 15 h (Figure 8), with residual estimation error remaining below the calculated RMS threshold despite external perturbations bounded by δ. Furthermore, the adaptive law (Equation (17)) ensures the global convergence capability of the estimation error, enabling the system to maintain significantly lower error variance than fixed-gain dedicated observer (DOS) schemes, which can deviate by up to 20% when the bioreactor operates outside the nominal linearization point.
- Although the bioreactor model is highly sensitive to kinetic and inhibition parameters, the proposed robust adaptive observer architecture is designed to mitigate the problem of parametric identifiability. Rather than attempting to identify each parameter in isolation, the gain update law *ℓ* (see Equation (17)) considers the combined impact of parametric uncertainties and external disturbances within the bounds of δ. This adaptability ensures that the estimation error converges on a neighborhood of the origin, even when the nominal parameters diverge from the actual ones. This is crucial for fault diagnosis: the observer “tunes” to the actual system dynamics, allowing only abrupt, large-magnitude deviations to disrupt the balance of the error dynamics (see Equation (16)), thus preventing parametric uncertainty from being mistaken for a sensor failure.
- The proposed FDD method is based on a full-order dedicated observer scheme whose detection capability relies on generating robust residuals $r=y-C\hat{x}$ that are analyzed using a moving root-mean-square (RMS) window. Unlike data-driven strategies, such as in [35,37], this approach does not require prior training with fault databases. Instead, it uses an adaptive law (Equation (17)) that absorbs noise and slow parametric variations. This ensures that the residual only exceeds the threshold $J_{th}$ in the event of genuine anomalies. The scheme demonstrates high sensitivity in identifying abrupt and bias faults almost instantaneously, while maintaining superior immunity to false alarms, thanks to the perturbation bound δ integrated into the design. However, the method has limitations in detecting extremely slow drifts, as the adaptive nature of the observer could assimilate such deviations as normal physiological changes of the bioprocess. This is a common challenge discussed in recent reviews by [36].
- The FDD scheme was validated through a systematic binary classification analysis. Each fault scenario (bias and drift) was evaluated by comparing the time-stamped fault injection with the residual response of the observer relative to the threshold σ. The “accuracy rate” was then calculated as the ratio of the duration of successful detection periods to the total duration of induced faults. This methodology confirms a diagnostic accuracy exceeding 90%; the remaining margin is attributed to the observers’ inherent convergence time and the filtering of measurement noise [36]. Recall TP/(TP+FN) and the F1-score: $2\cdot \frac{Prec\cdot Rec}{Prec+Rec}$ were calculated to evaluate the capability of the detection system and its robustness against false alarms, respectively. Due to the inherent class imbalance in bioprocesses, where nominal operation predominates, the F1-score was prioritized as the overall performance metric as it penalizes both false negatives, which compromise safety, and false positives resulting from process noise. Detection thresholds were selected using a statistical criterion based on the disturbance bound δ and the maximum RMS value under nominal conditions. This ensures that the reported metrics are statistically significant and repeatable across multiple experimental runs. This systematic approach enables more transparent validation than the black box models of [35] and ensures that the adaptive observer maintains high sensitivity, independent of the variance of the kinetic parameters described in [36].
- A programming algorithm associates the waste generated by each sensor failure case with the triggering of alarms, which completes the real-time monitoring system for use by the personnel supervising the process. We can therefore conclude that the fault detection and diagnosis mechanism based on the dedicated observer scheme (DOS) is fully applicable and efficient at detecting anomalies and malfunctions in installed measuring instruments [38,39,40].
  Additionally, metrics were calculated for RMS residual values and precision, recall rate, and overall accuracy for abrupt failures. Normalization of the data was necessary due to the feasibility, magnitude, and quantification of the variables. Figure 11 shows the evolution of the residual evaluation of the function in the presence of a biomass sensor failure (Case 1). Based on the metric, the RMS residual value exceeds the proposed threshold, indicating a sensor failure. However, this is not biochemically correct because the microorganism cannot reach those growth contractions due to inhibition by the type of operation, as well as by the reaction stoichiometry.
  Accuracy is a more adequate metric if the dataset is balanced, meaning there is an approximately equal number of examples in each class and false positives and false negatives have similar implications. The F1-score is a unified metric that is a weighted average of precision and recall, therefore encompassing both false positives and false negatives. In our case, the data range for these metrics is 93–97% on average (see Figure 12).
- The economic optimization achieved through the implementation of virtual sensors (based on state observers) in bioreactors is significant, especially for difficult-to-measure variables such as ethanol, substrate, biomass, and glucose. For example, these state variables require an online NIR/FTIR flow analyzer/spectrometer, which has an estimated acquisition cost of between %15,000 and %50,000 USD. The FDD approach eliminates the need for an invasive sensor that is prone to failure due to fouling or sterilization, thereby reducing maintenance costs and saving capital while avoiding the need for sterile sampling bypasses. In the fermentation industry specifically, a 2% improvement or a 15% reduction in batch time can quickly translate into a return on investment (ROI) for the observer in less than a year, given that the cost of software estimation is negligible compared with the total operating cost of an industrial bioreactor. Finally, it increases yield by 2–5% of total batch production. It enables an optimal fed-batch strategy by controlling the feed rate and reducing cycle time by 10–20%, thereby freeing up bioreactor capacity. It improves process robustness and enables the early detection of faults, reducing losses due to failed batches by 1–5% annually.
- Biological systems are highly sensitive, and changes in operating conditions (temperature, pH, dissolved oxygen, and nutrient levels) can cause deviations from nominal operating conditions. These deviations are known as “faults”. In addition, since bioprocesses frequently involve unknown faults due to a lack of measurements, undefined stoichiometry (which creates uncertainty in black box models), and precursor and metabolite concentrations being not always known in real time, unsupervised algorithms are favored, as they can identify and diagnose faults without the need for prior knowledge of their occurrence [9]. Therefore, it is crucial to define how a bioprocess normally operates so that a fault detection algorithm can be implemented. As industrial automation evolves and data collection intensifies, the use of machine learning (ML) for real-time industrial fault detection, compared with traditional rule-based systems, offers superior adaptability, real-time responsiveness, and improved performance in equipment, processes, and products [24,35,36,37,41].
- Research finally provides a fundamental basis for industrial scalability by demonstrating that diagnostic success depends not only on the hardware, but also on the diagnostic core’s ability to withstand changes in operating conditions. A key indicator of this transition is the Model Fidelity Metric (MFM), which suggests that if the observer maintains a residual estimation error of less than 5% at the laboratory scale, the system will tolerate the mass transfer fluctuations that are typical of large-volume tanks. While online sensors (e.g., biomass and substrate) are often unavailable in commercial plants, this study shows that adaptive law can compensate for this if integrated with virtual sensors that translate large-scale operating conditions into actionable data for the observer. Therefore, whereas purely data-driven approaches [35] are affected by batch-to-batch variability, our approach guarantees an F1-score of at least 0.90 by dynamically adjusting its estimation gains. This offers a viable technical solution to the heterogeneity and noise issues identified by [36] in real industrial settings.

## 6. Conclusions

The full-order robust adaptive observer structure will show good performance when estimating the states with the biomass signal as an available measurement. However, it was possible to develop model-based fault detection and diagnosis mechanisms using state observers. For the batch bioreactor, additive failures that could be present in the turbidity sensor and the $CO_{2}$ probe were taken into account, occurring individually or simultaneously within the process. The proposed dedicated observer scheme (DOS) mechanism was complemented with physical redundancy methods and the dynamics of the mathematical model, enabling both the isolation and estimation of faults in real time efficiently. In this way, the user achieves correct monitoring, guaranteeing accurate knowledge of the process that will enable early intervention in decision making in the event of malfunction of the measuring instruments.

Thus, effective fault detection and diagnosis (FDD) strategies based on dedicated observers have been obtained using the root-mean-square (RMS) value of the residual, a practical threshold for validation, and metrics such as the precision, recall rate, and overall accuracy of three diagnostic metrics for abrupt failures.

On the other hand, the monitoring system was implemented in real time in the bioreactor operating in batches, this time with the Labview™ platform. The turbidity sensor measurement (biomass) was associated with the adaptive observer input to estimate the state variables whose concentrations are unknown due to the absence of sensors: substrate, ethanol, and $CO_{2}$. In addition, the FDD mechanism was implemented to diagnose failures in the two online turbidity sensors used for physical redundancy. Having two sensors that measure biomass ensures that, if one malfunctions, the other provides continuity to the monitoring. This is fundamental to understanding concentrations that cannot be measured online. When failures occurred, the performance of the bioreactor degraded. However, applying the FDD methods based on observers, analytical models, and physical redundancies allows us to detect, isolate, and identify faults correctly.

Finally, an accuracy rate of over 90% was calculated based on successful detection across multiple fault scenarios presented. We compared the time taken for fault injection with the residual response time. This is sustained by robust LMI tuning, where the 10% margin accounts for observer convergence time and filtering of high-frequency noise.

## Figures and Tables

**Table 1:** A review of the application of observers to the process of alcoholic fermentation.

Observers	Expression	Measurable Variables	Estimated Variables
Robust Full Order (batch) [8]	$\dot{\hat{x}}$ *= (A − LC)* $\hat{x}$ *+ Bu + LCx*	Reaction temperature.	Triglyceride concentration, temperature.
Robust Full Order (continuous) [9,10,11]	$\dot{\hat{x}}$*= A*$\hat{x}$*+ Bu − L(Cx − C*$\hat{x}$)	$CH_{4},CO_{2}$	Substrate, biomass.
Adaptable Full Order (batch, continuous) [12]	$\dot{\hat{x}}$*= A*$\hat{x}$*+ Bu − L(t)(Cx − C*$\hat{x}$)	$CH_{4},CO_{2}$	Substrate, biomass.

**Table 2:** The parameters and units of measurement for batch operations.

Parameters	Value	Units
$\kappa_{1}$	0.05	h^−1^
$\kappa_{2}$	0.007	Lg^−1^ h^−1^
$\kappa_{3}$	0.02	h^−1^
$\kappa_{4}$	0.007	h^−1^
$\kappa_{5}$	1	Dimensionless
$\kappa_{6}$	1	Dimensionless
$\kappa_{7}$	0.01	Dimensionless
$\kappa_{8}$	0.001	Dimensionless
$\kappa_{9}$	500	g/L
$\kappa_{10}$	1	Lg^−1^ h^−1^
$\kappa_{11}$	0.01	h^−1^

**Table 3:** Estimation for different configurations of *C*.

Configuration *C*	Substrate	Biomass	Ethanol	${\mathrm{CO}}_{2}$
[1000]	•	•	•	•
[0100]	•	•	•	•
[0010]	•	•	•	∘
[0001]	•	•	∘	•

**Table 4:** Observers’ adaptive full order.

Simulation Characteristics	Values
Observer gain matrix	*ℓ* = [−120;−20;−152;−18]
Initial conditions of the system	*S* = 20 g/L, *X* = 0.5 g/L, Et = 0.43 g/L, $CO_{2}$ = 0.17 g/L
Initial conditions of the observer	*S* = 10 g/L, *X* = 0.4 g/L, Et = 1.5 g/L, $CO_{2}$ = 0.1 g/L

**Table 5:** Changes in system parameters.

Parameters	Parameter Values	Modified Parameter Values (Batch)
$\kappa_{1}$	0.05 h^−1^	0.1 h^−1^
$\kappa_{2}$	0.007 Lg^−1^ h^−1^	0.03 Lg^−1^ h^−1^
$\kappa_{3}$	0.02 h^−1^	0.05 h^−1^
$\kappa_{4}$	0.007 h^−1^	0.002 h^−1^
$\kappa_{5}$	1	2
$\kappa_{6}$	1	3
$\kappa_{11}$	1 h^−1^	1.5 h^−1^

**Table 6:** Gains of adaptive observers implemented in real time and initial conditions for bioreactor operating in batch.

Observers	Gains Matrix	Initial Conditions (g/L)
System observer.	ℓ=[−51;−20;−55;−22]	[20;0.4;0;0.1]
First observer of the bank.	ℓ=[−51;−20;−55;−22]	[20;0.4;0;0.1]
Second observer of the bank.	ℓ=[−0.2;−12;−128.5;−19.8]	[25;0.8;1.5;0.1]

## Data Availability

The original contributions presented in this study are included in the article. Further inquiries can be directed to the corresponding authors.

## Appendix A. Main Panel

In the main panel in Figure A1, there is a general and simple visualization of the state variables and the sensors connected in line with the process. In this case, there are two turbidity sensors, where the first of them is used as input to the robust observer who will estimate the concentrations of substrate, biomass, ethanol, and $CO_{2}$. The second turbidity sensor is used as input to the second state observer of the implemented FDD mechanism. The objective is to prove that the proposed DOS scheme truly works to isolate sensor failures, regardless of the process. For this case, some variations are made in the programming, but the logic followed is the same. In Section 1, a graph is shown with the estimation of each state variable and its representation according to the mathematical model. The first run was carried out without the occurrence of failures in the sensors. In Section 2, there is a real-time visualization of the output signals of each turbidity sensor, converted to biomass concentration. Figure A1 shows the graph of the output signals of each sensor when they are functioning correctly. In Section 3, the operation of the turbidity sensors is displayed, activating alarms in case of anomalies occurring in them. Each sensor has a pair of LED lamps associated with it. The ones on the left mark the status of the ON/OFF sensors to detect whether they are delivering a voltage signal or not. In addition, the lamps on the right display overload alarms associated with the values of each residue generated by the bank of observers. Finally, in Section 4 of this screen, the simulation control is shown, where the elapsed time in hours and the emergency stop button are indicated.

## References

[1] Erdogmus D., Genç A.U., Príncipe J.C. (2002). A neural network perspective to extended Luenberger observers. Meas. Control.

[2] Yang J., Zhong Q., Shi K., Zhong S. (2022). Co-design of observer-based fault detection filter and dynamic event-triggered controller for wind power system under dual alterable DoS attacks. IEEE Trans. Inf. Forensics Secur..

[3] Kumar M.A. (2011). Biosensors and Automation for Bioprocess Monitoring and Control.

[4] Zhou M., Wu Y., Wang J., Xue T., Raïssi T. (2024). Zonotopic state estimation and sensor fault detection for a wastewater treatment bioprocess. Int. J. Robust Nonlinear Control.

[5] Reyes S.J., Durocher Y., Pham P.L., Henry O. (2022). Modern sensor tools and techniques for monitoring, controlling, and improving cell culture processes. Processes.

[6] Rahul S.G., Chitra R., Kumar V.P., Abhishek P.H.S., Reddy B.O. (2022). Virtual Instrumentation Based Graphical User Interface for Fermentation Bioprocess Monitoring Using LabVIEW. Innovations in Mechanical Engineering: Select Proceedings of ICIME 2021.

[7] Alvarado-Santos E., Aguilar-López R., Neria-González M.I., Romero-Cortés T., Robles-Olvera V.J., López-Pérez P.A. (2023). A novel kinetic model for a cocoa waste fermentation to ethanol reaction and its experimental validation. Prep. Biochem. Biotechnol..

[8] Röbenack K. (2004). Computation of the observer gain for extended Luenberger observers using automatic differentiation. IMA J. Math. Control Inf..

[9] Luenberger D. (2003). An introduction to observers. IEEE Trans. Autom. Control.

[10] Li Y., Li H., Xiao G. (2023). Luenberger-like observer design and optimal state estimation of logical control networks with stochastic disturbances. IEEE Trans. Autom. Control.

[11] Kalman R.E. (1960). On the General Theory of Control Systems. IFAC Proc. Vol..

[12] García G., Moreno J.F., Bernal T., Posso F., Delgado-Noboa J. (2024). Kinetic modeling of Mead production. J. Am. Soc. Brew. Chem..

[13] Hernandez J., Medina R., Hernandez M. (2012). Instrumentation and Design of a Supervisory System for an Anaerobic Biodigestor. 2012 IEEE International Symposium on Alternative Energies and Energy Quality (SIFAE).

[14] Tenkolu G.A., Kuffi K.D., Gindaba G.T. (2024). Optimization of fermentation condition in bioethanol production from waste potato and product characterization. Biomass Convers. Biorefin..

[15] Mercorelli P. (2024). Recent advances in intelligent algorithms for fault detection and diagnosis. Sensors.

[16] Escanciano I.A., Ladero M., Santos V.E., Blanco Á. (2023). Development of a simple and robust kinetic model for the production of succinic acid from glucose depending on different operating conditions. Fermentation.

[17] Talebnia F., Karakashev D., Angelidaki I. (2010). Production of bioethanol from wheat straw: An overview on pretreatment, hydrolysis and fermentation. Bioresour. Technol..

[18] Iswarya P., Manikandan K. (2024). Algorithms for Fault Detection and Diagnosis in Wireless Sensor Networks Using Deep Learning and Machine Learning—An Overview. 2024 10th International Conference on Communication and Signal Processing (ICCSP).

[19] Du P., Abdel Jabbar N.M., Wilhite B.A., Kravaris C. (2025). Fault Diagnosis in Chemical Reactors with Data-Driven Methods. Ind. Eng. Chem. Res..

[20] Du S., Wang W., Fu H., Wan X. (2023). Fault Detection and State Estimation in Automatic Control. Appl. Sci..

[21] Kim Y., Chica-Carrillo E.C., Lee H.J. (2024). Microfabricated sensors for non-invasive, real-time monitoring of organoids. Micro Nano Syst. Lett..

[22] Rösner L.S., Walter F., Ude C., John G.T., Beutel S. (2022). Sensors and techniques for on-line determination of cell viability in bioprocess monitoring. Bioengineering.

[23] Jennita J.P., Velvizhi G. (2024). Co-fermentation exploiting glucose and xylose utilizing thermotolerant S. cerevisiae of highly lignified biomass for biofuel production: Statistical optimization and kinetic models. Biocatal. Agric. Biotechnol..

[24] Aviles J.D., Torres-Zuniga I., Villa-Leyva A., Vargas A., Buitron G. (2022). Experimental validation of an interval observer-based sensor fault detection strategy applied to a biohydrogen production dark fermenter. J. Process Control.

[25] Perdukova D., Fedor P., Sobek M., Bačík J. (2024). A Simple Linearization Method of Nonlinear Systems Based on Fuzzy Logic. IEEE Access.

[26] Tamayo Roman E.D., Ordaz Oliver J.P., López Pérez P.A. (2025). Design and simulation of a full-order robust adaptive observer for monitoring of the biogas process. Int. J. Chem. React. Eng..

[27] Martínez-Sibaja A., Alvarado-Lassman A., Posada-Gómez R., Mendez-Contreras J.M., Gonzalez-Sanchez B.E., Sandoval-González O. (2013). Dedicated observer scheme for fault diagnosis and isolation in instruments of an anaerobic reactor. Procedia Technol..

[28] Garcia E.A., Frank P.M. (1996). Analysis of a class of dedicated observer schemes to sensor fault isolation. UKACC International Conference on Control’96 (Conf. Publ. No. 427).

[29] Aguilar López R., Ruiz Camacho B., Neria-González M.I., Rangel E., Santos O., López Pérez P.A. (2017). State estimation based on nonlinear observer for hydrogen production in a photocatalytic anaerobic bioreactor. Int. J. Chem. React. Eng..

[30] Chimmiri V., Karri R. (2022). Optimal state and parameter estimation for fault detection and diagnosis of a nonlinear batch beer fermentation process. Optimal State Estimation for Process Monitoring, Fault Diagnosis and Control.

[31] Díaz-Choque M., Chaccara-Contreras V., Aquije-Cárdenas G., Atoche-Wong R., Villanueva-Acosta V., Gamarra-Bustillos C., Samanamud-Loyola O. (2022). Supervision, control, and data acquisition system of a heat exchanger. Indon. J. Electr. Eng. Comput. Sci..

[32] Hernández-Melchor D.J., Camacho-Pérez B., Ríos-Leal E., Alarcón-Bonilla J., López-Pérez P.A. (2020). Modelling and multi-objective optimization for simulation of hydrogen production using a photosynthetic consortium. Int. J. Chem. React. Eng..

[33] Hernández-Melchor D.J., Camacho-Pérez B., Ríos-Leal E., Alarcón-Bonilla J., López-Pérez P.A. (2018). Experimental and kinetic study for lead removal via photosynthetic consortia using genetic algorithms to parameter estimation. Environ. Sci. Pollut. Res..

[34] Perez-Perez E.J., Fragoso-Mandujano J.A., Guzmán-Rabasa J.A., González-Baldizón Y., Flores-Guirao S.K. (2024). ANFIS and Takagi–Sugeno interval observers for fault diagnosis in bioprocess system. J. Process Control.

[35] Hematillake D., Freethy D., McGivern J., McCready C., Agarwal P., Budman H. (2022). Design and optimization of a penicillin fed-batch reactor based on a deep learning fault detection and diagnostic model. Ind. Eng. Chem. Res..

[36] Aghaee M., Mishra A., Krau S., Tamer I.M., Budman H. (2024). Artificial intelligence applications for fault detection and diagnosis in pharmaceutical bioprocesses: A review. Curr. Opin. Chem. Eng..

[37] Lim S.J., Son M., Ki S.J., Suh S.I., Chung J. (2023). Opportunities and challenges of machine learning in bioprocesses: Categorization from different perspectives and future direction. Bioresour. Technol..

[38] Rodríguez A.E., Torres J., Pérez J.R., Domínguez A.R., Luna R., Flores G. (2016). Robust state estimation in presence of parametric uncertainty by nl-pi observers. an application to continuous microbial cultures. IEEE Lat. Am. Trans..

[39] Rodriguez-Mata A.E., Bustos-Terrones Y., Gonzalez-Huitrón V., Lopéz-Peréz P.A., Hernández-González O., Amabilis-Sosa L.E. (2020). A fractional high-gain nonlinear observer design—Application for rivers environmental monitoring model. Math. Comput. Appl..

[40] Fay C.D., Corcoran B., Diamond D. (2023). Green IoT event detection for carbon-emission monitoring in sensor networks. Sensors.

[41] Windmann S. (2022). Data-driven fault detection in industrial batch processes based on a stochastic hybrid process model. IEEE Trans. Autom. Sci. Eng..

